# Magnitude and trends in cervical cancer at Mbarara Regional Referral Hospital in South Western Uganda: Retrospective analysis of data from 2017–2022

**DOI:** 10.1371/journal.pgph.0002848

**Published:** 2024-01-19

**Authors:** Rogers Kajabwangu, Francis Bajunirwe, Jonathan Izudi, Joel Bazira, Yarine Farjardo, Frank Ssedyabane, Henry Mark Lugobe, Joy Muhumuza, Musa Kayondo, Stuart Turanzomwe, Thomas C. Randall, Joseph Ngonzi

**Affiliations:** 1 Department of Obstetrics and Gynaecology, Faculty of Medicine, Mbarara University of Science and Technology, Mbarara, Uganda; 2 Department of Community Health, Mbarara University of Science and Technology, Mbarara, Uganda; 3 Department of Medical Microbiology, Mbarara University of Science and Technology, Mbarara, Uganda; 4 Department of Medical Laboratory Science, Faculty of Medicine, Mbarara University of Science and Technology, Mbarara, Uganda; 5 Department of Obstetrics and Gynecology, Gynecological Oncology Division, Massachusetts General Hospital, Boston, Massachusetts, United States of America; University of Nebraska Medical Center Pharmacy Practice: University of Nebraska Medical Center College of Pharmacy, UNITED STATES

## Abstract

High-income countries have documented a significant decline in the incidence and mortality of cervical cancer over the past decade but such data from low and middle-income countries such as Uganda is limited to ascertain trends. There is also paucity of data on the burden of cervical cancer in comparison to other gynaecologic malignancies and there is a likelihood that the incidence might be on the rise. To describe the current trends and magnitude of cervical cancer in comparison to other gynaecological malignancies histological types, we conducted a retrospective records review of charts of patients admitted with gynaecological malignancies on the gynaecological ward of Mbarara Regional Referral Hospital (MRRH) between January 2017 and December 2022. Of 875 patients with gynaecological malignancies admitted to the MRRH in the 6-year review period, 721 (82.4%) had cervical cancer. Patients with cervical cancer were significantly older than those with other gynaecological malignancies: (50.2±11.5 versus 43.8± 15.0 respectively, p<0.001). Between 2017 and 2022, cervical cancer rates increased by 17% annually compared to other gynaecological cancers (OR:1.17; 95% CI 1.06–1.28, p = 0.0046), with the majority of patients of cervical cancer patients (92.7%, n = 668) having squamous cell carcinoma. Most patients (87.9%, n = 634) had late-stage disease (stage 2 and above) and were referred to the Uganda Cancer Institute for chemoradiation. These results imply that there is a need to scale up screening services and other preventive measures such as vaccination against human papilloma virus.

## Background

Globally, cervical cancer is a major public health problem and is the fourth most common malignancy among women [1]. Estimates from the most recent data show nearly half a million new cervical cancer cases and 307,000 deaths occurred in Sub-Saharan Africa in 2020, representing 80% and 90% of the global new cervical cancer cases and deaths respectively [2].

Compared to high-income countries, low and middle-income countries have not recorded decreasing trends in the incidence and mortality of cervical cancer with existing evidence, although inconclusive, suggesting a steady rise [3, 4].

There is limited up-to-date data on the trends of cervical cancer in resource-limited settings [5]. In addition, national and regional cancer registries occasionally miss critical data [6, 7]. However, one study conducted a decade ago reported cervical cancer as the most prevalent gynaecological cancer at Mbarara Regional Referral Hospital (MRRH) in southwestern Uganda, averaging 107 cases per annum [8]. The study further showed that cervical cancer constitutes one in four of all reported cancer cases in the hospital and more than seven in 10 (74%) of all gynaecological cancers, with the majority of the patients presenting to the hospital in an advanced cervical cancer stage [8]. It is important to review more recent data and understand the current trends in disease burden. With cervical cancer screening coverage in Uganda standing below 10% over the years [9], cervical cancer mortality might likely worsen due to delayed access to effective care and treatment [10]. Therefore, this study describes the magnitude and trends in the proportion of cervical cancer relative to other gynaecological malignancies between 2017 and 2022 and describes the histological types and stage distribution at diagnosis over the same period at Mbarara Regional Referral Hospital in South-western Uganda.

Our study is significant in contributing to the literature on cervical cancer, an increasing problem among women in Uganda, and informing the regional and national cervical cancer control, prevention, and treatment services to reduce its morbidity and mortality. Another significance of our study is the demonstration of whether current cervical cancer control and prevention efforts are achieving the desired outcomes or not. Our study also provides evidence for targeting future cervical cancer control measures in Uganda and similar settings.

## Materials and methods

### Study setting

The study was conducted at the Gynaecology ward of Mbarara Regional Referral Hospital (MRRH), the main referral hospital in southwestern Uganda and the facility serves at least 10 districts with an estimated catchment population of 6.3 million people [11]. The hospital also serves patients from neighboring Tanzania, Rwanda, Burundi, and the Eastern Democratic Republic of Congo. MRRH is the main teaching hospital for Mbarara University of Science and Technology Medical School. The Gynaecology Ward has a bed capacity of 40 patients and is run by six gynaecologists, with one gynaecological oncologist plus several residents, intern doctors, and nurses. The hospital operates a daily cervical cancer clinic where screening for cervical cancer and treatment for premalignant cervical cancer lesions is performed. On average, about 20 patients attend the cervical cancer clinic daily. The hospital also runs a histopathology laboratory which is operated by three pathologists and several other staff. Patients seen in the cervical cancer clinic with suspicious cervical lesions undergo cervical biopsy and the tissue is sent to this laboratory for histopathological analysis. Patients with confirmed cervical cancer are transferred to the Gynaecology Ward for examination under anaesthesia and clinical staging.

Following staging, those with early-stage cancer i.e. stage 2A and below, undergo appropriate surgical procedures while those with advanced cancer are referred to the Uganda Cancer Institute at Mulago National Referral Hospital, in the capital Kampala, for chemoradiotherapy.

### Study design, population, and sampling procedure

We performed a retrospective review of medical records of patients admitted with gynaecological malignancies on the gynaecological ward at MRRH between January 2017 and December 2022. Between 1^st^ March 2023 and 31^st^ March 2023, we retrieved all available medical records (census) for the review period provided the patients had a diagnosis of gynaecological malignancy. For all the other cancers except gestational trophoblastic neoplasia, only patient charts with histological confirmation of gynaecological cancer were included in the study. For gestational trophoblastic neoplasia, we included patients whose charts had laboratory evidence of a persistently elevated serum beta-hCG as this is diagnostic [12, 13]. Medical charts that were missing histological results or serum Beta-hCG in the case of gestational trophoblastic neoplasia were excluded. The study was approved by the Mbarara University of Science and Technology Research Ethics Committee (Reference number: MUST-2022-576) and registered by the National Council for Science and Technology (Reference number: HS3053ES). The ethics committee provided a waiver of informed consent to access medical records as it was not logistically feasible to trace and obtain consent from all the participants. Administrative clearance was obtained from the Mbarara Regional Referral Hospital management before the study commencement. Although the research team had access to all the records, information such as names that could identify the participants was not extracted to preserve confidentiality.

### Data collection and variables

We developed a data extraction tool in REDCap and used it to extract data from the patient’s charts. We extracted the following variables: age, district of residence, parity, diagnosis, date of diagnosis, and the type of malignancy. For patients with cervical cancer, we extracted additional variables namely, histology, stage at diagnosis, nature and duration of symptoms, and initial treatment modality. Late stage at diagnosis was defined as a presentation at stage IIb and above according to the International Federation of Gynaecology and Obstetrics (FIGO) staging system [14].

### Data management and analysis

We downloaded the data from REDCap into a Microsoft Excel spreadsheet (Microsoft Office Professional Plus 2013, version 15.0.4675.1003, Microsoft Inc, Redmond, Washington, USA) and imported it into Stata version 17 (StataCorp LLC, College Station, Texas, United States) for analysis. We summarized categorical data using frequencies and percentages, and numerical data using means and standard deviations if normally distributed otherwise the median and interquartile range (IQR) was used if skewed. We tested differences in the proportions of the outcome variable with categorical data using the Chi-square for large cell counts (≥5) or Fisher’s exact test for smaller cell counts (<5). For mean differences with numerical data, we used the Student’s t-test for normally distributed data or the Wilcoxon-rank sum test for skewed numerical data. To determine the magnitude of cervical cancer among gynecological cancers, the total number of patients with cervical cancer over the study period was divided by the total number of patients with gynecological cancers and expressed as a percentage. The proportion of each cancer was computed and displayed in a bar graph. To demonstrate the trend of cervical cancer, the annual proportions of cervical cancer were compared with that of other gynecological cancers over six years using a line graph.

To demonstrate whether the risk of cervical cancer increased over the years compared with the other gynecological malignancies, we performed a generalized additive model as trends were non-linear and reported the odds ratio with a corresponding 95% confidence interval.

## Results

### Participant recruitment

**Fig 1:**
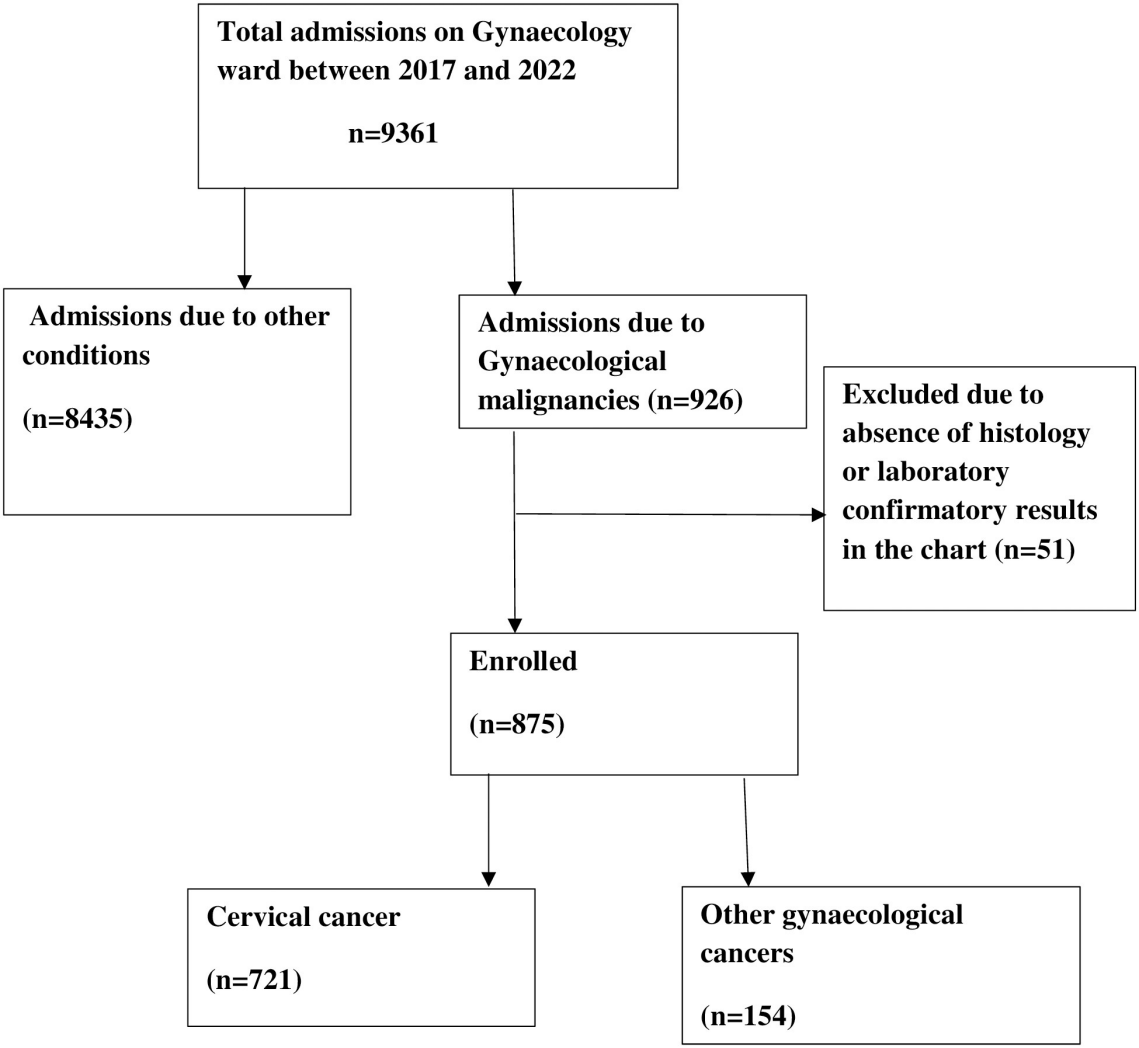
Participant recruitment chart.

We reviewed charts for 9361 patients admitted to the gynaecology ward between 2017 and 2022. Of these, 926 patients had a diagnosis of gynaecological cancer. Only 875 women had evidence of a histological diagnosis of gynaecological cancer or persistently elevated serum beta-hCG in the case of gestational trophoblastic neoplasia and were enrolled. We excluded 51 patients because they lacked evidence of histological diagnosis or serum beta-hCG in the case of gestational trophoblastic neoplasia and the details are shown in Fig 1.

### Demographic characteristics of patients with gynaecological malignancies at Mbarara Regional Referral Hospital

On average, patients with cervical cancer were significantly older compared to those with other gynaecological malignancies: (50.2±11.5 versus 43.8± 15.0 respectively, p<0.001). Patients with cervical cancer had a higher parity (≥5) compared to those with other gynecological malignancies. There was no difference regarding residence and HIV status between women who had cervical cancer and those who had other gynaecological malignancies (Table 1).

**Table 1:** Demographic characteristics of patients with gynaecological malignancies at Mbarara Regional Referral Hospital between January 2017 and December 2022.

Characteristic	Level	Overall, (n = 875, 100%)	Cervical cancer, (n = 721, 82.4%)	Other gynecological cancers, (n = 154,17.6%)	p-value
Age	15–24	16 (1.8)	3 (0.4)	13 (8.4)	<0.001
	25–34	93 (10.6)	57 (7.9)	36 (23.4)	
	35–44	181 (20.7)	155 (21.5)	26 (16.9)	
	≥45	585 (66.9)	506 (70.2)	79 (51.3)	
	Mean age (SD)	49.05 (12.4)	50.2(11.5)	43.8 (15.0)	<0.001
District of residence	Mbarara	225 (25.7)	186 (25.8)	39 (25.3)	0.900
	Outside Mbarara	650 (74.3)	535 (74.2)	115 (74.7)	
HIV status	Negative	463 (52.9)	382 (53.0)	81 (52.6)	0.170
	Positive	205 (23.4)	176 (24.4)	29 (18.8)	
	Unknown	207 (23.7)	163 (22.6)	44 (28.6)	
Parity	0 to 1	60 (6.9)	27 (3.7)	33 (21.4)	<0.001
	2 to 4	255 (29.1)	194 (26.9)	61 (39.6)	
	> = 5	560 (64)	500 (69.3)	60 (39.0)	
Year of admission	2017	152 (17.4)	128 (17.8)	44 (28.6)	<0.001
	2018	193 (22.0)	164 (22.7)	29 (18.8)	
	2019	182 (20.8)	140 (19.4)	42 (27.3)	
	2020	118 (13.5)	109 (15.1)	9 (5.8)	
	2021	112 (12.8)	76 (10.5)	16 (10.4)	
	2022	118 (13.5)	104 (14.4)	14 (9.1)	

### The magnitude of cervical cancer at Mbarara Regional Referral Hospital between January 2017-December 2022

**Fig 2:**
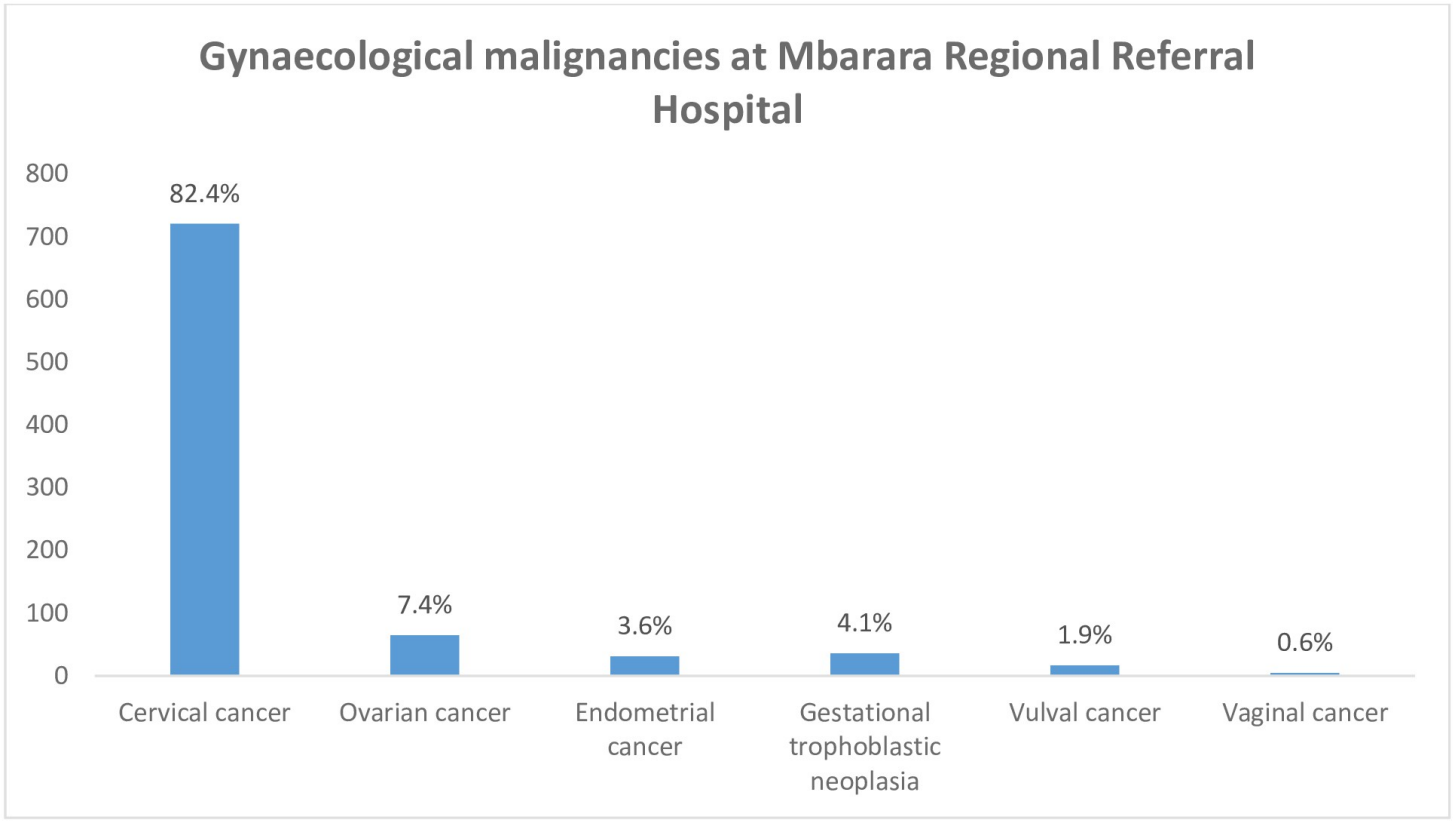
The magnitude of cervical cancer among women admitted with gynaecological malignancies at Mbarara Regional Referral Hospital between 2017 and 2022.

Fig 2 shows the magnitude of cervical cancer among patients with gynaecological malignancies at Mbarara regional referral hospital between 2017–2022. 82.4% (721 /875) of these patients had cervical cancer while the rest had other gynaecological malignancies: ovarian cancer (7.4%, 65/875), gestational trophoblastic neoplasia (4.1%, 36/875), endometrial cancer (3.5%, 31/875), vulval cancer (1.9%, 17/875), and vaginal cancer (0.6%, 5/875).

### Trends of cervical cancer at MRRH

**Fig 3:**
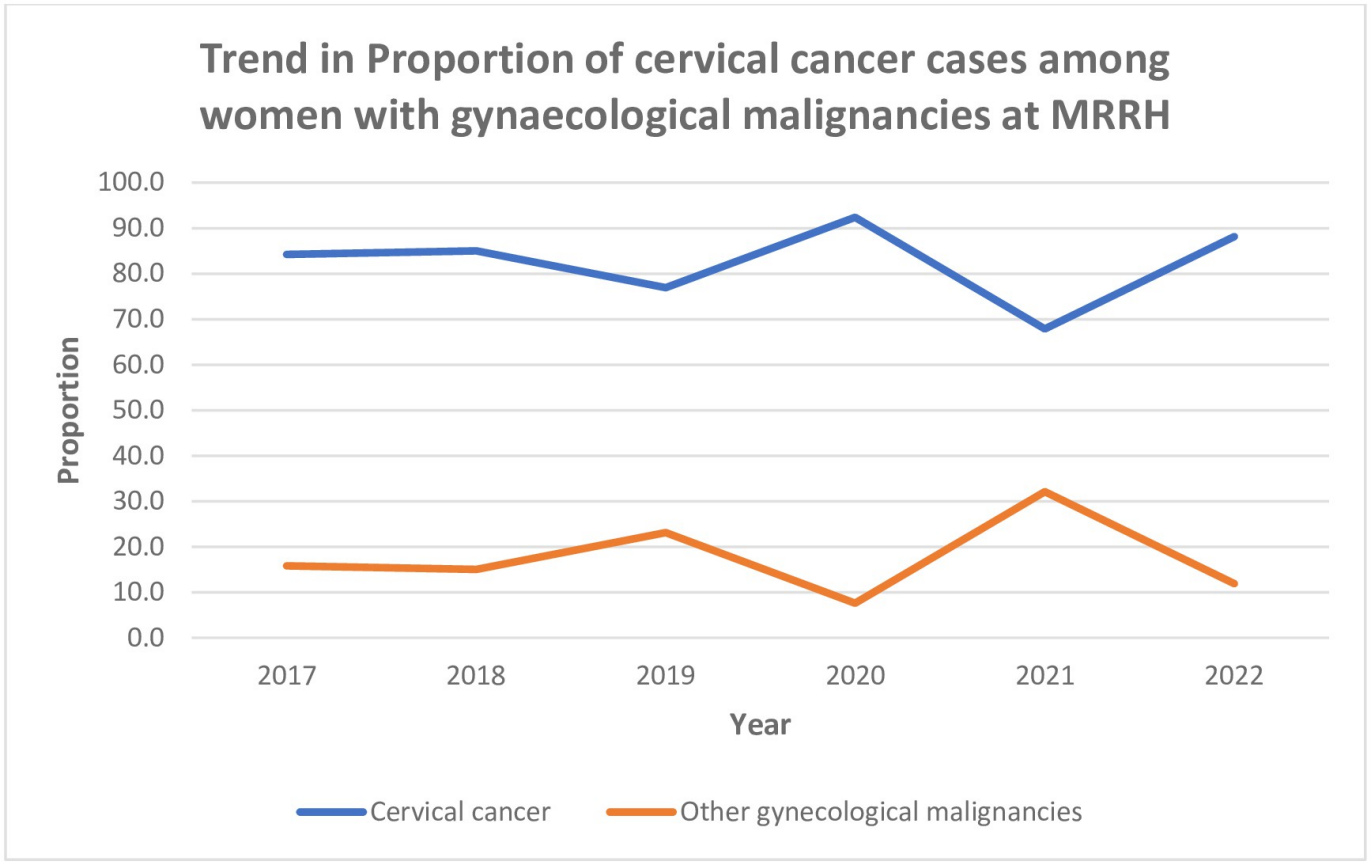
The trend in the proportion of cervical cancer cases at MRRH.

Fig 3 shows the trends in the proportion of cervical cancer between 2017 and 2022. The odds of cervical cancer increased by 17% for every additional year compared to other gynaecological cancers (OR, 1.17; 95% CI 1.06–1.28, p = 0.0046).

### Clinical presentation of patients with cervical cancer at Mbarara Regional Referral Hospital

The majority of the patients (92.7%) had squamous cell carcinoma and presented with vaginal bleeding which lasted between 2 and 6 months. Most of the patients had late-stage disease (stage 2 and above) at diagnosis and were subsequently referred to the Uganda Cancer Institute for chemoradiation and the details are shown in Table 2 below.

**Table 2:** Clinical presentation of patients with cervical cancer at Mbarara Regional Referral Hospital (n = 721).

Characteristic	Level	Frequency	Percentage
**Histology**	Squamous cell carcinoma	668	92.7
	Adenocarcinoma	44	6.1
	Adenosquamous carcinoma	9	1.2
**Symptoms**	Vaginal bleeding	497	68.9
	Foul-smelling vaginal discharge	333	46.2
	Abdominal pain	409	56.7
	Hematuria	17	2.4
**Symptom duration (months)**	≤1	81	11.2
	2–6	409	56.7
	7–12	150	20.8
	>12	81	11.2
**FIGO stage at diagnosis**	Stage I	49	6.8
	Stage IIA	38	5.3
	Stage IIB	190	26.4
	Stage III	363	50.4
	Stage IV	81	11.2
**Stage type at diagnosis**	Early stage	87	12.1
	Late stage	634	87.9
**Treatment given** *	Surgery	85	11.8
	Blood transfusion	195	27.0
	Antibiotics	546	75.5
	Analgesics	500	69.3
	Referral	454	63.0

Note:

* The number under treatments exceeds the number of patients because some patients received more than one treatment mode.

## Discussion

In this retrospective review of medical records, we describe the magnitude and trend of cervical cancer at Mbarara Regional Referral Hospital between 2017 and 2022 and the common histological types and stage distribution at diagnosis.

The study shows that cervical cancer is the leading gynaecological cancer at MRRH, constituting about 82% of admissions for gynaecological malignancies. Our data show that on average, 120 women are admitted with cervical cancer annually at MRRH, which is consistent with previous reports in Uganda and other African countries, indicating a high burden of cervical cancer among women in low-income countries [5, 8, 15, 16].

Between 2017 and 2022, the odds of cervical cancer compared to other gynaecological cancers increased by 17% for every additional year. This increase is consistent with previous findings a decade ago [8] and implies that there are still gaps in the implementation of the strategic plan for cervical cancer prevention and control in Uganda [17]. For example, the uptake of cervical cancer screening in Uganda currently stands at only 4.8% [18]. Other preventive measures for cervical cancer such as human papillomavirus vaccination are grossly underused [19], making it unlikely that there will be a reduction in the number of cervical cancer cases among Ugandan women. On the contrary, this number is likely to increase tremendously as projected by the WHO [20].

There was a drop in the number of patients admitted with cervical cancer at MRRH in 2021. This could be attributed to the COVID-19 pandemic disruptions in the utilization of healthcare services, particularly for non-communicable diseases which have been demonstrated in other studies [21].

In this study, most cervical cancer patients presented in the late stage of the disease, which is a common challenge in resource-limited settings [22, 23]. The late presentation might be explained by poor health-seeking behaviour and a lack of awareness combined with inequitable access to cervical cancer prevention and control services [24–26]. Late stage at diagnosis reduces the chance of survival and increases the risk of death [27], and it is a central factor in driving the high cervical cancer mortality rate in low-income countries [2, 28].

Moreover, chemoradiation, which is the treatment for late-stage cervical cancer is unavailable in most of the health facilities in low-income countries [29]. In Uganda for example, there’s only one center for chemoradiation, which is in the capital of the country [22]. This implies that patients diagnosed with late-stage cervical cancer have to travel very long distances to access this service.

We found squamous cell carcinoma (SCC) as the most common histological cervical cancer type accounting for about 93% of the cases. This proportion of squamous cell carcinoma in comparison with other types of cervical cancer, particularly adenocarcinoma, is higher than that previously estimated at 75% [30, 31]. Compared to adenocarcinoma, squamous cell carcinoma is easier to prevent with conventional screening methods like visual inspection methods and cytology since its lesions grow outwards, towards the ectocervix [32, 33]. A scaling up of screening services using the available methods is therefore likely to reduce the burden of cervical cancer in this region in the long run.

### Strengths and limitations

This study provides important data on the trend over an extended period, measures the magnitude of cervical cancer in southwestern Uganda, and strengthens the efforts for screening and other prevention programs. Our study has some weaknesses. As with most studies using medical records, some charts were missing critical data like histology results and were excluded. Our results likely underestimated the magnitude of the burden of disease.

## Conclusion

The burden of cervical cancer at Mbarara Regional Referral Hospital is high compared to other gynaecologic malignancies, with an increase every year. Squamous cell carcinoma is the commonest histological type of cervical cancer and most patients present in the late stage of the disease. There is a need to scale up screening services and other preventive measures to reduce the burden of cervical cancer.

## Supporting information

S1 Data Data set-Mbarara cervical cancer. The variable labels are indicated in the first row.
(XLS)

## References

[1] Arbyn M, Weiderpass E, Bruni L, de Sanjosé S, Saraiya M, et al. (2020) Estimates of incidence and mortality of cervical cancer in 2018: a worldwide analysis. The Lancet Global Health 8: e191–e203. doi: 10.1016/S2214-109X(19)30482-6 31812369 PMC7025157

[2] Sung H, Ferlay J, Siegel RL, Laversanne M, Soerjomataram I, et al. (2021) Global cancer statistics 2020: GLOBOCAN estimates of incidence and mortality worldwide for 36 cancers in 185 countries. CA: a cancer journal for clinicians 71: 209–249. doi: 10.3322/caac.21660 33538338

[3] Buskwofie A, David-West G, Clare CA (2020) A review of cervical cancer: incidence and disparities. Journal of the National Medical Association 112: 229–232. doi: 10.1016/j.jnma.2020.03.002 32278478

[4] Akinyemiju TF (2012) Socio-economic and health access determinants of breast and cervical cancer screening in low-income countries: analysis of the World Health Survey. PloS one 7: e48834. doi: 10.1371/journal.pone.0048834 23155413 PMC3498259

[5] Jedy-Agba E, Joko WY, Liu B, Buziba NG, Borok M, et al. (2020) Trends in cervical cancer incidence in sub-Saharan Africa. British journal of cancer 123: 148–154. doi: 10.1038/s41416-020-0831-9 32336751 PMC7341858

[6] Shimakawa Y, Bah E, Wild CP, Hall AJ (2013) Evaluation of data quality at the Gambia national cancer registry. International Journal of Cancer 132: 658–665. doi: 10.1002/ijc.27646 22618962

[7] Joko-Fru WY, Jedy-Agba E, Korir A, Ogunbiyi O, Dzamalala CP, et al. (2020) The evolving epidemic of breast cancer in sub-Saharan Africa: Results from the African Cancer Registry Network. International Journal of Cancer 147: 2131–2141. doi: 10.1002/ijc.33014 32306390

[8] Mayanja R (2016) Cervical Cancer at Mbarara Regional Referral Hospital: Magnitude, Trends, Stages at Presentation, Impact of Acetic Acid Screening and the Need for Radiotherapy Services. Journal of Health, Medicine and Nursing 27.

[9] Bruni L, Serrano B, Roura E, Alemany L, Cowan M, et al. (2022) Cervical cancer screening programmes and age-specific coverage estimates for 202 countries and territories worldwide: a review and synthetic analysis. The Lancet Global Health 10: e1115–e1127. doi: 10.1016/S2214-109X(22)00241-8 35839811 PMC9296658

[10] Chongsuwat T, Ibrahim AO, Evensen AE, Conway JH, Zwick M, et al. (2023) Health facility assessments of cervical cancer prevention, early diagnosis, and treatment services in Gulu, Uganda. PLOS Global Public Health 3: e0000785. doi: 10.1371/journal.pgph.0000785 36962762 PMC10021907

[11] Uganda Bureau of Statistics (2020) Uganda Bureau of Statistics 2020 statistical abstract. Kampala. https://www.ubos.org/wp-content/uploads/publications/11_2020STATISTICAL__ABSTRACT_2020.pdf

[12] Oncology F (2002) Committee: FIGO staging for gestational trophoblastic neoplasia 2000. FIGO Oncology Committee. Int J Gynaecol Obstet 77: 285–287. 12065144 10.1016/s0020-7292(02)00063-2

[13] Ngan H, Bender H, Benedet J, Jones H, Montruccoli G, et al. (2003) Gestational trophoblastic neoplasia, FIGO 2000 staging and classification. International Journal of Gynecology & Obstetrics 83: 175–177. doi: 10.1016/s0020-7292(03)90120-2 14763174

[14] Shi J, Dong Y, Jiang W, Qin F, Wang X, et al. (2022) MRI-based peritumoral radiomics analysis for preoperative prediction of lymph node metastasis in early-stage cervical cancer: a multi-center study. Magnetic resonance imaging 88: 1–8. doi: 10.1016/j.mri.2021.12.008 34968703

[15] Bruni L, Barrionuevo-Rosas L, Albero G, Serrano B, Mena M, et al. (2017) ICO information centre on HPV and cancer (HPV information centre). Human papillomavirus and related diseases in the world Summary Report 27.

[16] International agency for research on cancer I (2018) IARC. GLOBOCAN 2018 Cervical Cancer Facts Sheet.

[17] MOH (2010) Strategic plan for cervical cancer prevention and control in Uganda 2010–2014. Ministry of Health Kampala. 70 p.

[18] Ndejjo R, Mukama T, Musabyimana A, Musoke D (2016) Uptake of cervical cancer screening and associated factors among women in rural Uganda: a cross sectional study. PloS one 11: e0149696. doi: 10.1371/journal.pone.0149696 26894270 PMC4760951

[19] Isabirye A, Mbonye M, Asiimwe JB, Kwagala B (2020) Factors associated with HPV vaccination uptake in Uganda: a multi-level analysis. BMC Women’s Health 20: 1–11. 32660461 10.1186/s12905-020-01014-5 PMC7359563

[20] World Health Organization (2013) Projections of mortality and causes of death, 2015 and 2030.

[21] Formenti B, Gregori N, Crosato V, Marchese V, Tomasoni LR, et al. (2022) The impact of COVID-19 on communicable and non-communicable diseases in Africa: a narrative review. Le Infezioni in Medicina 30: 30. doi: 10.53854/liim-3001-4 35350264 PMC8929725

[22] Nakisige C, Schwartz M, Ndira AO (2017) Cervical cancer screening and treatment in Uganda. Gynecologic oncology reports 20: 37–40. doi: 10.1016/j.gore.2017.01.009 28275695 PMC5331149

[23] Kantelhardt EJ, Moelle U, Begoihn M, Addissie A, Trocchi P, et al. (2014) Cervical cancer in Ethiopia: survival of 1,059 patients who received oncologic therapy. The oncologist 19: 727–734. doi: 10.1634/theoncologist.2013-0326 24951611 PMC4077439

[24] Modibbo FI, Dareng E, Bamisaye P, Jedy-Agba E, Adewole A, et al. (2016) Qualitative study of barriers to cervical cancer screening among Nigerian women. BMJ open 6: e008533. doi: 10.1136/bmjopen-2015-008533 26754174 PMC4716205

[25] Tsu VD, Levin CE (2008) Making the case for cervical cancer prevention: what about equity? Reproductive Health Matters 16: 104–112. doi: 10.1016/S0968-8080(08)32411-2 19027628

[26] Ginsburg O, Bray F, Coleman MP, Vanderpuye V, Eniu A, et al. (2017) The global burden of women’s cancers: a grand challenge in global health. The Lancet 389: 847–860. doi: 10.1016/S0140-6736(16)31392-7 27814965 PMC6191029

[27] Wright JD, Matsuo K, Huang Y, Tergas AI, Hou JY, et al. (2019) Prognostic performance of the 2018 International Federation of Gynecology and Obstetrics cervical cancer staging guidelines. Obstetrics and gynecology 134: 49. doi: 10.1097/AOG.0000000000003311 31188324 PMC7641496

[28] Momenimovahed Z, Mazidimoradi A, Maroofi P, Allahqoli L, Salehiniya H, et al. (2023) Global, regional and national burden, incidence, and mortality of cervical cancer. Cancer Reports 6: e1756. doi: 10.1002/cnr2.1756 36545760 PMC10026270

[29] Abdel-Wahab M, Bourque J-M, Pynda Y, Iżewska J, Van der Merwe D, et al. (2013) Status of radiotherapy resources in Africa: an International Atomic Energy Agency analysis. The lancet oncology 14: e168–e175. doi: 10.1016/S1470-2045(12)70532-6 23561748

[30] Small W Jr, Bacon MA, Bajaj A, Chuang LT, Fisher BJ, et al. (2017) Cervical cancer: a global health crisis. Cancer 123: 2404–2412. doi: 10.1002/cncr.30667 28464289

[31] Poole J, Patnick J (2012) Profile of cervical cancer in England. Trent Cancer Registry.

[32] Zappa M, Visioli C, Ciatto S, Iossa A, Paci E, et al. (2004) Lower protection of cytological screening for adenocarcinomas and shorter protection for younger women: the results of a case–control study in Florence. British journal of cancer 90: 1784–1786. doi: 10.1038/sj.bjc.6601754 15150597 PMC2409750

[33] Mitchell H, Medley G, Gordon I, Giles G (1995) Cervical cytology reported as negative and risk of adenocarcinoma of the cervix: no strong evidence of benefit. British journal of cancer 71: 894–897. doi: 10.1038/bjc.1995.172 7710961 PMC2033741

